# Editorial: Medical and Industrial Applications of Microfluidic-Based Cell/Tissue Culture and Organs-on-a-Chip

**DOI:** 10.3389/fbioe.2019.00151

**Published:** 2019-06-26

**Authors:** Qasem Ramadan, Massimo Alberti, Martin Dufva, Yi-Chung Tung

**Affiliations:** ^1^Agency for Science, Technology and Research (A^*^STAR), Singapore, Singapore; ^2^Department of Health Technology, Technical University of Denmark, Kongens Lyngby, Denmark; ^3^Research Center for Applied Sciences, Academia Sinica, Taipei, Taiwan

**Keywords:** cell culture, microfluidics, *in vitro*, organs-on-a-chip, drug, toxicology

Cell culture on Petri dish remains the gold standard in basic research for screening drug candidates during one of the most expensive and lengthy industrial product development. However, the artifactual environment and the oversimplified structure of this one-cell-type culture system do not mimic the *in vivo* dynamic nature and heterogeneous architecture. Hence, it is not able to answer increasingly emerging biological questions. Animal models, on the other hand, provide systemic *in vivo* settings. However, despite the wealth of knowledge acquired from these models, many details of human pathogenesis cannot be addressed because of the differences between animal and human immune responses in preclinical studies vs. clinical trials (Mestas and Hughes, 2004; Zschaler et al., 2014). Costs associated with the failure in predicting the toxicity and efficacy of a drug candidate, particularly in the clinical trial stages, have become overwhelmingly expensive (Mullard, 2018). Therefore, the pharmaceutical industry needs to develop more predictive tools that reduce the chance of failure.

The past two decades have seen a rapid growth of microfluidics-based cell culture technology, with the ultimate aim to boost the development of fundamental bioscience and pharmaceutics. The versatile functionality and excellent spatiotemporal control over micro-environmental elements in microfluidic-based cell culture open up wide possibilities for tissue engineering and next-generation drug discovery. A remarkable development that is recently emerged is the ability to co-culture various type of cells in an integrated fluidic network to emulate a specific human tissue or organ simplified structure and function, called organs on a chip (OOC). A plethora of microfluidic-based culture models have been developed; however, the adaptation of this technology to address biological questions still scattered.

Here, we have assembled papers by contributors from prominent research labs that discuss fundamental questions and highlight the recent development in this emerging and rapidly advancing technology. There are five unique reviews, one perspective paper, and six original research papers in this issue that present a critical assessment of recent literature and demonstrate new advances in the field.

Avendano et al. discuss the effect of the physical microenvironment remodeling associated with cancer progression on mass transport in the tumor interstitial space. They examine the application and future opportunities of microfluidic models to better identify the physiochemical mediators of the mass transport. To maintain *in vivo*-like mass transfer and cell-cell communication *in vitro*, heterotypic cell co-culture is essential to mimic tissues and organs. Achieving such a heterotypic structure requires controlling the cell adhesion with desired patterns, Yaman et al. review the application of magnetic force-based cell manipulation in microfluidic devices and the potential use of this technique in guidance of cells into a specific location and creating heterotypic cellular structures in 2D and 3D organization.

Three papers in this collection focus on the brain tissue. *In vitro* modeling of the brain tissue, including the blood-brain barrier, and related diseases requires a high level of biological and fluidic design, as it is critical to replicate the complex network of different brain regions. Frimat and Luttge emphasize the advantages of combining engineered microsystems with stem cell (hiPSC) technology to improve the performances of brain-on-a-chip devices and their clinical relevance. Badiola-Mateos et al. address the challenges of modeling the neuromuscular circuit and recommend the combination of microfluidic systems, hiPSC and 3D culture to create patient-specific and reliable *in vitro* models. Qiao et al. discuss recent work on organoid-on-a-chip (Wang et al., 2018) as an alternative human-relevant neurodevelopmental model and its potential use for understanding the effects of prenatal nicotine exposure in the early stages of embryonic development, eliminating ethical concerns regarding human clinical trials in smoking pregnant women.

Spheroids and organoids have become popular tools for oncology and for basic and translational patient-specific tissue research. In order to grow large tissue models and organoids, the cells within the artificial tissue need continuous feeding through an *in vivo*-like capillary network. Mimicking the human vasculature, which can be viewed as a complex closed-loop perfusion system, represents one of the major *in vitro* challenges. Grebenyuk and Ranga reviewed the recent progress in generating *in vitro* vascularization. To date, nearly all fabrication techniques fall short to keep cells in 3D tissues alive during the slow fabrication process. Organ-on-a-Chip technology would enable the incorporation of the right cells at the right place, ultimately achieving perfusable organoids. An unconventional approach is reported by Bottaro et al. to demonstrate physical vein models by populating the surfaces of a PDMS mold with endothelial cells to simulate blood vessels and characterize the fluidic behavior of sclerosing foams.

Another significant challenge, especially when reconstructing epithelial tissues *in vitro*, is to obtain a realistic barrier function which is reviewed by Torras et al. Various epithelial barriers have been demonstrated *in vitro*, including lungs, intestine and skin. Bioprinting and photolithography are used for producing engineered complex basement membrane, recapitulate the properties of native epithelial tissues and provide robust predictive models.

Besides providing physiologically relevant cellular structure and fluidic environment, microfluidic devices also allow simulating the micro- and macro-mechanical features in human tissue. For instance, Felder et al. examined the effect of cyclic mechanical stress induced by respiratory motions, which was mimicked using a thin membrane, on alveolar wound repair. Recent breakthroughs in microbiology and medical sciences have highlighted the importance that the microbiota has on human health. Shin et al. demonstrated an *in vitro* anoxic-oxic interface of obligate anaerobic gut microbiome and human intestinal epithelial cells co-culture in a microfluidic chip and highlighted the importance of the host-microbiome crosstalk to the homeostasis of gastrointestinal functions.

Among all *in vitro* models, liver models play a central role in drug discovery. Accurate *in vitro* modeling of the liver is challenging because different hepatic functions are specific to the hepatocytes' spatial location within a liver lobule, which translate in different levels of gene expression and metabolic competence. Supported by a mathematical model, Tomlinson et al. used a Quasi Vivo system to create a zonated *in vitro* liver model in which primary rat hepatocytes cultured in three chambers are exposed to varying oxygen tension. This system provides a more accurate evaluation of pharmacological interventions at a zone-specific level.

Finally, in order to become widely accepted, these new culture systems need to overcome several drawbacks, such as the ability to control the concentrations of soluble factors, especially drugs. Lohasz et al. takes advantage of a tubing-free microfluidic device architecture to construct a microtissue culture system to generate gradual *in vivo*-like substance exposure profiles. The device not only eliminates the tedious interconnections for microfluidic device but also provides a great capability to control compound gradients in spatiotemporal domains.

This exciting collection of papers represent an excellent fit into the “Frontiers in Bioengineering and Biotechnology” with state-of-the-art contributions and critical reviews on the new advances in this growing field, with an emphasis on the interface between the technological advancements and high impact applications. We are delighted to be a part of this effort and witness the rapid expanding of the organ-on-a-chip technology toward opening the door to a variety of scientific and commercial avenues.

## Author Contributions

All authors listed have made a substantial, direct and intellectual contribution to the work, and approved it for publication.

### Conflict of Interest Statement

The authors declare that the research was conducted in the absence of any commercial or financial relationships that could be construed as a potential conflict of interest.
